# *Comamonas kerstersii* Bacteremia of Unknown Origin

**DOI:** 10.1155/2022/1129832

**Published:** 2022-03-27

**Authors:** Kassie Rong, Johan Delport, Fatimah AlMutawa

**Affiliations:** ^1^Department of Physiology and Pharmacology, Western University, London, ON, Canada; ^2^Department of Pathology and Laboratory Medicine, Western University, London, ON, Canada; ^3^London Health Science Center, London, ON, Canada

## Abstract

*Comamonas kerstersii* (*C. kerstersii*) is a Gram-negative bacillus abundant in the environment and rarely implicated in human disease. Previously considered nonpathogenic, its scarcity in literature may be partly due to the unreliability of past phenotypic tests used for its identification. In recent years, the development of matrix-assisted laser desorption ionization-time of flight (MALDI-TOF) mass spectrometry (MS) has enabled fast and accurate laboratory identification of *C. kerstersii*. Since the first report of human infection in 2013, several others have emerged, with most cases involving peritoneal infection. Here, we present a rare case of *C. kerstersii* bacteremia in an 82-year-old male patient. With no clear predisposing conditions, the source of his infection is unclear. We accompany this report with a review of *C. kerstersii* bacteremia cases found in the literature.

## 1. Introduction

*Comamonas kerstersii* (*C.kerstersii*) is a nonfermenting, Gram-negative bacillus widely distributed in the environment. It belongs to a genus first described in 1985 as including the single species *Comamonas terrigena* [1]. In 2003, the identification of genotypic and phenotypic differences led to the separation of *C. terrigena* into three distinct species: *C. terrigena*, *C. aquatica*, and *C. kerstersii* [2]. The genus now includes 22 species [3], most of which were isolated from environmental sources [4]. *C. kerstersii* is known to be motile, urease negative, and oxidase positive and can reduce nitrate. Although *C. kerstersii* is found abundantly in soil and water, it has rarely been implicated in human infections. Cases may be underreported due to the difficulty of identifying and differentiating members of *Comamonas* by phenotypic tests [5] and due to some microbial identification databases not containing the species [6]. In recent years, matrix-assisted laser desorption ionization-time of flight (MALDI-TOF) mass spectrometry (MS) has enabled the rapid and accurate diagnosis of *C. kerstersii* infections [7]. Following the first report of severe infection in 2013, other cases have been published, most of which involve peritoneal infections. Here, we report a case of bacteremia caused by *C. kerstersii* in an 82-year-old male patient for whom the source of infection is unclear. We also review the cases of *C. kerstersii* bacteremia found in the literature.

## 2. Case Presentation

An 82-year-old gentleman presented to the emergency department in March 2021 with chief complaints of chills and rigours. His medical history included type 2 diabetes with diabetic neuropathy and dyslipidemia. The patient is not known to have HIV. He had previously been diagnosed with colon tubular and tubulovillous adenomas and invasive moderately differentiated squamous cell cancer of the right upper malar cheek, which had been completely excised. His home medications included tamsulosin, metformin, and ezetimibe.

At presentation, the patient reported no cough, urinary symptoms, or abdominal pain. However, he had been constipated for a few days, which was unusual for him. While in the ER, he spiked a fever of 38.6°C and became tachycardic at 120 bmp. He was stabilized and sent home after blood and urine samples were collected. On March 6, the patient was called back to the hospital due to a positive blood culture for Gram-negative bacilli. At readmission, he was awake and alert with no acute distress. Cardiovascular examination showed bilateral mild pitting edema. His blood pressure was 160/87 mmHg. The patient's abdomen was soft and distended but nontender in all quadrants, with no masses or organomegaly and no guarding or peritoneal signs. Chest X-rays showed clear lungs and pleural spaces and stable cardiomediastinal contour. Abdominal X-rays showed no free air, no significant bowel dilatation or air-fluid levels, and a moderate fecal load throughout his large bowel.

SARS-CoV-2, influenza A, influenza B, and RSV PCRs were done, and all were negative. Two blood culture sets were collected from two different sites. Both sets have aerobic and anaerobic bottles. Growth was detected at 1 and 2 days on the BACTEC system. Bacteria from the patient's blood culture were identified by MALDI-TOF as *Comamonas kerstersii.* His urine culture was negative. MicroScan antibiotic susceptibility test results are presented in Table 1. In addition to the table, *C. kerstersii* was susceptible to amikacin, ceftriaxone, cefotaxime, cefepime, and aztreonam based on CLSI M100 breakpoints for other non-enterobacterales [8]. The patient was diagnosed with bacteremia of unknown origin and admitted to internal medicine. Piperacillin-tazobactam was started for broad-spectrum coverage before the final susceptibility report.

The patient's bloodwork showed leukocyte count of 7.7 × 10 ^9^/L with 7.1 × 10^9^/L neutrophils, 1.3 × 10^9^/L lymphocytes and 0.1 × 10^9^/L for both monocyte and eosinophil. Sodium and potassium levels were normal. CRP was not done. He was prescribed one week of intravenous ceftriaxone (1 g/day) at home. The treating team elected to follow that with oral amoxicillin-clavulanic acid for one week as the patient was doing well and the organism was susceptible to piperacillin and ticarcillin-clavulanate (tested in the lab but not reported). At a follow-up appointment one week after discharge, the patient appeared to be doing well, and repeated blood cultures were negative. Cardiovascular examination was normal except for mild edema in the lower extremities, and abdominal examination was benign. His blood pressure was 148/82 mmHg. He was seen again at a two-week follow-up after discharge and was doing well clinically with no complaints.

## 3. Discussion

As stated previously, *C. kerstersii* has rarely been associated with infections in humans despite its abundance in the environment. As such, it had long been considered nonpathogenic [9]. Only in recent years have cases of severe infection have been described in the literature. In 2013, Almuzara et al. published the first four reports of *C. kerstersii* in humans, all of which were intra-abdominal infections [6]. Identification was attempted with several methods, including the standard phenotypic tests proposed by Wauters et al., the VITEK 2 Compact System, and MALDI-TOF MS along with PCR to confirm the results. The VITEK database does not contain *C. kerstersii* and may misidentify the species as *Comamonas testosteroni* [6]. MALDI-TOF MS has enabled the accurate and rapid identification of *C. kerstersii* and has been employed in all subsequent reports of the infection. Prior to this technology, clinical laboratories struggled to precisely identify and differentiate *Comamonas* species with phenotypic tests [5].

In the published literature, we found 25 cases of *C. kerstersii* infection, among which the main predisposing conditions were appendicitis [6, 7, 11, 12], peritonitis (often secondary to perforated appendicitis) [12], and perforated colon [6, 12]. The site of infection was most often peritoneal [6, 7, 9, 11, 12], with only three reported cases of bacteremia [7, 10, 13] and one each of psoas abscess [12] and urinary tract infection [3]. A likely source of peritoneal infection is the translocation of bacteria from the gut following environmental exposure [7]. This may also be a possible source in cases of bacteremia as *C. kerstersii* is a versatile organism capable of thriving under a variety of conditions.

Table 2 summarizes this current patient along with the three cases of *C. kerstersii* bacteremia found in the literature. Previous reports suggest a possible source of infection to be translocation of bacteria from the intestinal tract. Opota et al. describe a patient diagnosed with diverticulosis who reported drinking river water, a possible environmental source of the pathogen [7]. Along with *C. kerstersii*, the gut commensal *Bacteroides fragilis* was also identified from the patient's blood culture, supporting the theory of gut translocation. In two other reported cases of bacteremia, appendicitis was a predisposing condition, and the source of infection was likely appendicular [10, 13]. It has been posited that *C. kerstersii* may be commensal with bacteria in the appendix and digestive tract [10].

This current case is unusual as the patient had no identifiable predisposing conditions for his bacteremia. Neither his abdominal symptoms nor past medical history could be linked with a source of infection. Whether his constipation could have contributed to his bacteremia is not clear. Nevertheless, a complete recovery was achieved. Antimicrobial tests reveal that *C. kerstersii* is susceptible to a wide range of antibiotics. Based on this limited number of reports, the prognosis following infection is favourable. We emphasize that *C. kerstersii* infections may be underreported in literature due to past difficulties with its identification. While it was once thought nonpathogenic, the emergence of MALDI-TOF has enabled reliable identification of *C. kerstersii*, and it is now considered a possible opportunistic bacterium [10, 12].

## 4. Review of Published Cases

## Figures and Tables

**Table 1:** Antibiotic susceptibility of *Comamonas kerstersii*.

Drug	Interpretation	MIC (microgram/ ml)
Ceftazidime	S	**≤1**
Ciprofloxacin	R	**>2**
Gentamicin	S	**2**
Imipenem	S	**≤0.5**
Meropenem	S	**≤1**
Piperacillin/tazobactam	S	**≤4**
Tobramycin	S	**2**

MIC, minimal inhibitory concentration. S, susceptible. R, resistance.

**Table 2:** Clinical and microbiologic characteristics of *Comamonas kerstersii* bacteremia in humans.

Age	Sex	Clinical presentation	Underlying conditions	Predisposing conditions	Pathogens identified	Antibiotic treatment	Outcome	Reference
65	M	Abdominal pain, vomiting, diarrhea, fever, chills	Diabetes	Diverticulosis, ingested river water	*Comamonas kerstersii, Bacteroides fragilis*	Ciprofloxacin, then imipenem-cilastatin after identification	Recovered	[6]
31	M	Abdominal pain, nausea, vomiting, fever	None reported	Perforated appendicitis with abdominal abscess, acute peritonitis	*Comamonas kerstersii*	Cefuroxime axetil, then cefuroxime and metronidazole at discharge	Complete recovery	[10]
16	M	Abdominal pain, fever, nausea, vomiting	None reported	Acute appendicitis, appendicular abscess	*Comamonas kerstersii*	Ampicillin-sulbactam and metronidazole, then piperacillin-tazobactam after identification	Recovered	[13]
82	M	Chills, rigours, constipation, fever	Type 2 diabetes, diabetic neuropathy, dyslipidemia, colon tubular adenoma, tubulovillous adenoma	None	*Comamonas kerstersii*	Piperacillin-tazobactam, then ceftriaxone and amoxicillin-clavulanic acid at discharge	Recovered	Present case

## Data Availability

No extra data available for this case report.

## References

[1] De Vos P., Kersters K., Falsen E. (1985). 1962 Comamonas Davis and Park 1962 gen. nov, nom. rev. emend, and Comamonas terrigena Hugh 1962 sp. nov, nom. rev. *International Journal of Systematic Bacteriology*.

[2] Wauters G., De Baere T., Willems A., Falsen E., Vaneechoutte M. (2003). Description of Comamonas aquatica comb. nov. and Comamonas kerstersii sp. nov. for two subgroups of Comamonas terrigena and emended description of Comamonas terrigena. *International Journal of Systematic and Evolutionary Microbiology*.

[3] Almuzara M., Cittadini R., Estraviz M. L., Ellis A., Vay C. (2018). First report of *Comamonas kerstersii* causing urinary tract infection. *New Microbes and New Infections*.

[4] Wisplinghoff H., Cohen J, Powderly W. G, Opal S. M (2017). Pseudomonas spp., acinetobacter spp. and miscellaneous gram-negative bacilli. *Infectious Diseases*.

[5] Jiang X., Liu W., Zheng B. (2018). Complete genome sequencing of Comamonas kerstersii 8943, a causative agent for peritonitis. *Scientific Data*.

[6] Almuzara M. N., Cittadini R., Vera Ocampo C. (2013). Intra-abdominal infections due to Comamonas kerstersii. *Journal of Clinical Microbiology*.

[7] Opota O., Ney B., Zanetti G., Jaton K., Greub G., Prod’hom G. (2014). Bacteremia caused by comamonaskerstersii in a patient with diverticulosis. *Journal of Clinical Microbiology*.

[8] Clsi (2020). *Performance Standard for Antimicrobial Susceptibility Testing*.

[9] Liu X.-j., Qiao X.-w., Huang T.-m., Li L., Jiang S.-p. (2020). Comamonas kerstersii bacteremia. *Medecine et Maladies Infectieuses*.

[10] Zhou Y.-h., Ma H.-x., Dong Z.-y., Shen M.-h. (2018). Comamonas kerstersii bacteremia in a patient with acute perforated appendicitis. *Medicine*.

[11] Biswas J. S., Fitchett J., O’Hara G. (2014). Comamonas kerstersii and the perforated appendix. *Journal of Clinical Microbiology*.

[12] Almuzara M., Barberis C., Veiga F. (2017). Unusual presentations of *Comamonas kerstersii* infection. *New Microbes and New Infections*.

[13] Palacio R., Cabezas L., Cornejo C., Seija V. (2020). Bacteriemia por Comamonas kerstersii en un joven con apendicitis aguda. *Revista chilena de infectología*.

